# Lymphadenectomy and chemotherapy are effective treatments for patients with 2023 international federation of gynecology and obstetrics stage IIC-high risk endometrial cancer in Japan

**DOI:** 10.1007/s10147-024-02647-4

**Published:** 2024-11-09

**Authors:** Yoshinori Tani, Keiichiro Nakamura, Masae Yorimitsu, Noriko Seki, Mie Nakanishi, Hironori Itou, Miyuki Shimizu, Dan Yamamoto, Etsuko Takahara, Hisashi Masuyama

**Affiliations:** 1 Department of Obstetrics and Gynecology, Okayama University Graduate School of Medicine, Dentistry and Pharmaceutical Sciences, Okayama, Japan; 2 Department of Obstetrics and Gynecology, Hiroshima City Hiroshima Citizens Hospital, Hiroshima, Japan; 3 Department of Obstetrics and Gynecology, Japanese Red Cross Society Himeji Hospital, Hyogo, Japan; 4 Department of Obstetrics and Gynecology, Kagawa Prefectural Central Hospital, Kagawa, Japan; 5 Department of Obstetrics and Gynecology, National Hospital Organization Iwakuni Clinical Center, Yamaguchi, Japan; 6 Department of Obstetrics and Gynecology, Kagawa Rosai Hospital, Kagawa, Japan; 7 Department of Obstetrics and Gynecology, National Organization Fukuyama Medical Center, Hiroshima, Japan; 8 Department of Obstetrics and Gynecology, Fukuyama City Hospital, Hiroshima, Japan

**Keywords:** Endometrial cancer, FIGO 2023, Stage IIC high risk, Lymphadenectomy, Chemotherapy

## Abstract

**Background:**

In early-stage endometrial cancer (EC), the treatment of aggressive histological subtypes (endometrioid carcinoma grade 3, serous carcinoma, clear-cell carcinoma, undifferentiated carcinoma, mixed carcinoma, and carcinosarcoma) is controversial. We aimed to investigate the treatment of patients with International Federation of Gynecology and Obstetrics (FIGO) stage IC and stage IIC EC according to the 2023 classification.

**Methods:**

We retrospectively identified patients with FIGO 2023 stage IC, IIC-intermediate risk (IIC-I), and IIC-high risk (IIC-H) EC who underwent adjuvant therapy or observation after surgery at eight medical institutions from 2004 to 2023. Progression-free survival (PFS) and overall survival (OS) were evaluated using Kaplan–Meier estimates and univariate and multivariate analyses.

**Results:**

The PFS and OS were significantly worse in patients with FIGO 2023 stage IIC-H EC than in those with FIGO 2023 stage IIC-I EC (PFS: p = 0.008 and OS: p = 0.006). According to the FIGO 2023 stage IIC-H classification, lymphadenectomy and chemotherapy resulted in better prognoses regarding both PFS and OS (p < 0.001 for both) than other treatments. Our findings suggest that lymphadenectomy and chemotherapy effectively reduced vaginal stump and lymph node metastases in FIGO 2023 stage IIC-H EC (p < 0.001 and p = 0.008, respectively). Furthermore, in the multivariate analysis, not undergoing lymphadenectomy or chemotherapy were independent predictors of recurrence and poor prognoses in patients with FIGO 2023 stage IIC-H EC (p < 0.001 and p = 0.031, respectively).

**Conclusion:**

Lymphadenectomy and chemotherapy resulted in better prognoses regarding both recurrence and survival in patients with FIGO 2023 stage IIC high-risk EC.

## Introduction

Endometrial cancer (EC) is the most common gynecological malignancy in the United States, with an estimated 67,880 new cases diagnosed in 2024 [1]. In addition to endometrioid carcinoma, aggressive histologic subtypes such as serous, clear-cell, undifferentiated, mixed, carcinosarcoma, and other unusual types are increasing annually in Japan [2].

The International Federation of Gynecology and Obstetrics (FIGO) staging classification for EC (FIGO 2023) has been revised to include histopathological and molecular genetic information, in addition to traditional anatomical progression, marking a major shift toward improving prognostic predictability and individualized treatment. The FIGO 2023 staging analysis also divided the histological subtypes into typical endometrioid carcinoma and aggressive histological types, with aggressive histological types consisting of endometrioid carcinoma G3, serous carcinoma, clear-cell carcinoma, undifferentiated carcinoma, mixed carcinoma, carcinosarcoma, and other unusual types [3]. Previously, the stage IA aggressive histologic type of EC (FIGO 2009) was defined as < 50% myometrial invasion; however, in the FIGO 2023 stage classification, it was classified as IC or IIC depending on whether there was invasion of the myometrium. In addition, if there is an aggressive histological type of myometrial invasion, the disease is classified as stage IIC according to the FIGO 2023 progression stage of EC.

Currently, treatment in Japan is planned according to the guidelines of the Japanese Society of Gynecologic Oncology (JSGO) [2]. However, there have been no reports on the current clinical status of early-stage aggressive histological subtypes of EC in Japan according to the FIGO 2023 stage classification. Therefore, we aimed to investigate the clinical status of early-stage aggressive histological EC subtypes in Japan.

## Patients and methods

### Study population

This multicenter retrospective study included 473 patients with FIGO 2023 (stage classification IC or IIC) EC treated between January 2004 and December 2023. The study was approved by the Institutional Ethics Committee of Okayama University (approval number: 2311-023). All procedures were performed in accordance with the relevant ethical standards and institutional ethics committee regulations. From the medical records, we extracted clinical and pathological data regarding FIGO 2023 stage classification, histology, myometrial invasion, lymphovascular space (LVS) involvement, peritoneal cytology, date of progression, date of the last follow-up visit, and patient status at the last visit. All patients underwent total laparotomic or laparoscopic hysterectomies, bilateral salpingo-oophorectomies, or omentectomies with or without pelvic or para-aortic lymphadenectomies. Pelvic lymph node dissection included the right and left common iliac nodes; external and internal iliac nodes; and supra-inguinal, obturator, sacral, and parametrial nodal chains. Para-aortic lymph node dissection included nodes located at the bifurcation of the aorta and the renal vein. Adjuvant chemotherapy consisted of paclitaxel (175–180 mg/m^2^ infused over 3 h) and carboplatin (dose required to achieve an area under the concentration-time curve of 5–6). Patients who chose to undergo radiotherapy or neoadjuvant chemotherapy were also excluded. Treatment was provided according to the JSGO guidelines, but lymph node dissection and chemotherapy were not performed due to consideration of PS, age, and complications. Treatment decisions were left to the discretion of the facility.

### Statistical analysis

Statistical analyses were performed using the Mann–Whitney U test for comparison with controls. Progression-free survival (PFS) and overall survival (OS) were analyzed using the Kaplan–Meier method. Differences between recurrence and survival curves were examined using the log-rank test. Univariate and multivariate analyses were performed using the Cox proportional hazards model to determine the factors that predicted PFS and OS after adjusting for known prognostic factors. Analyses were performed using SPSS software (version 26.0; SPSS Inc., Chicago, IL, USA). All p values < 0.05 were considered statistically significant.

## Results

### Patient characteristics

We investigated the distribution of each clinical characteristic, including patient age, body mass index (BMI), histology, myometrial invasion, LVS involvement, peritoneal cytology, hysterectomy, lymphadenectomy, para-aortic lymphadenectomy, treatment, recurrence, and death, based on EC of FIGO 2023 stage classifications of IC and IIC. FIGO 2023 IC includes intermediate-risk patients in the JSGO guideline (no myometrial invasion but aggressive histology), and FIGO 2023 IIC includes both intermediate and high-risk patients (intermediate risk; endometrioid carcinoma G3 and myometrial invasion < 1/2, high risk; endometrioid carcinoma G3 and myometrial invasion ≧1/2 or other histologies [serous carcinoma, clear-cell carcinoma, undifferentiated carcinoma, mixed carcinoma, carcinosarcoma, and other unusual types] with any myometrial invasion). Therefore, we divided the patients into three groups: IC, IIC-intermediate risk (IIC-I), and IIC-high risk (IIC-H). The median patient age was 66.0 years (range 25–92 years). The median patient BMI was 23.8 kg/m^2^ (range 15.1–44.5 kg/m^2^). Data regarding EC of FIGO 2023 stage classification IC, IIC-I, and IIC-H, age, BMI, histology, myometrial invasion, LVS involvement, peritoneal cytology, hysterectomy, lymphadenectomy, para-aortic lymphadenectomy, treatment, recurrence, and death are shown in detail in Table 1.

**Table 1:** Patient and tumor characteristics

Baseline characteristics						
Stage	IC		IIC-I		IIC-H	
	Numbers	(%)	Numbers	(%)	Numbers	(%)
Age (years)						
< 40	0	0	0	0	3	0.9
40–49	6	8.2	5	7.5	15	4.5
50–59	15	20.5	25	37.3	72	21.6
60–69	22	30.1	17	25.4	102	30.6
70–79	26	35.6	15	22.4	93	27.9
≥ 80	4	5.5	5	7.5	48	14.4
BMI (kg/m^2^)						
< 18.5	5	6.8	5	7.5	35	10.5
18.5–24.9	44	60.3	35	52.2	205	61.6
25.0–29.9	17	23.3	19	28.4	61	18.3
30.0–34.9	5	6.8	5	7.5	25	7.5
35.0–39.9	1	1.4	3	4.5	5	1.5
≥ 40.0	1	1.4	0	0	2	0.6
Histology						
Endometrioid adenocarcinoma G3	16	21.9	67	100	79	23.7
Carcinosarcomas	11	15.1	–	–	97	29.1
Clear-cell carinoma	9	12.3	–	–	17	5.1
Mixed type carcinoma	5	6.8	–	–	42	12.6
Serous carcinoma	31	42.5	–	–	86	25.8
Undifferentiated carcinoma	1	1.4	–	–	12	3.6
Myometrial invasion						
≤ 50%	–	–	67	100	165	49.5
> 50%	–	–	–	–	168	50.5
LVS involvement						
Absent	70	95.9	38	56.7	188	56.5
Present	3	4.1	29	43.3	145	43.5
Peritoneal cytology						
Absent	68	93.2	65	97	299	89.8
Present	5	6.8	2	3	34	10.2
Hysterectomy						
Laparoscopy	12	16.3	10	14.9	21	6.3
Laparotomy	61	83.7	57	85.1	312	93.7
Lymphadnectomy						
Laparoscopy	8	11	6	9	7	2.1
Laparotomy	39	53.4	61	91	165	97.9
Para-aortic lymphadenectomy						
Laparoscopy	3	4.1	0	0	2	0.6
Laparotomy	20	27.4	22	32.8	115	34.5
Treatment						
Lymphadnectomy (−); Chemotherapy (–)	12	16.4	11	16.4	67	20.1
Lymphadnectomy ( +); Chemotherapy (–)	9	12.3	1	1.5	33	9.9
Lymphadnectomy (–); Chemotherapy ( +)	14	19.2	14	20.9	69	20.7
Lymphadnectomy ( +); Chemotherapy ( +)	38	52.1	41	61.2	161	48.3
Recurrence						
Absent	67	91.8	59	88.1	240	72.1
Present	6	8.2	8	11.9	93	27.9
Death						
Absent	70	95.9	66	98.5	287	86.2
Present	3	4.1	1	1.5	46	13.8

*BMI* body mass index, *LVI* lympho-vascular space

### Sites of recurrence

There were six patients (8.2%) with recurrence in the IC group, eight patients (11.9%) with recurrence in the IIC-I group, and 93 patients (27.9%) with recurrence in the IIC-H group. In the IC group, there were four patients with a single recurrence and two with multiple recurrences. In the IIC-I group, there were seven patients with a single recurrence and one with multiple recurrences. In the IIC-H group, there were 64 patients with a single recurrence and 29 with multiple recurrences. Of the patients in the IC group with recurrences, one had vaginal stump metastases (VSM), three had disseminated metastases (DM), four had lymph node metastases (LNM), and one had hematogenous metastases (HM). Of the patients in the IIC-I group with recurrences, four had VSM, two had DM, two had LNM, and two had HM. Of the patients in the IIC-H group with recurrences, 23 had VSM, 30 had DM, 29 had LNM, and 30 had HM. The recurrence sites are shown in detail in Table 2.

**Table 2:** Patient and tumor characteristics

Sites of recurrence						
Stage	IC (73)		IIC-I (67)		IIC-H (333)	
	Numbers	%	Numbers	%	Numbers	%
Recurrence						
Single	4	5.5	7	10.4	64	19.2
Multiple	2	2.7	1	1.5	29	8.7
Recurrence site						
Vaginal stump metastases (VSM)	1	1.4	4	6	23	6.9
Disseminated metastases (DM)	3	4.1	2	3	30	9
Pelvic metastases	1	1.4	1	1.5	11	3.3
Extrapelvic metastases	2	2.7	1	1.5	19	5.7
Lymph node metastases (LNM)	4	5.5	2	3	29	8.7
Pelvic metastases	3	4.1	1	1.5	19	5.7
Extrapelvic metastases	1	1.4	1	1.5	14	4.2
Hematogenous metastases (HM)	1	1.4	2	3	30	9
Lung metastases	1	1.4	1	1.5	24	7.2
Liver metastases	0	0	1	1.5	1	0.3
Bone metastases	0	0	0	0	5	1.5

### Histology and site of recurrence

In the IC group, six patients relapsed, of which one had endometrioid carcinoma G3, two had carcinosarcoma, and three had serous carcinoma. In the IIC-M group, 93 patients relapsed, of which 26 had endometrioid carcinoma G3, 21 had carcinosarcoma, 16 had mixed-type carcinoma, 21 had serous carcinoma, and 8 had clear-cell and undifferentiated carcinoma. Mixed-type carcinoma had a significantly higher incidence of multiple recurrences than carcinosarcoma (p = 0.044) and had a significantly higher incidence of LNM than endometrioid carcinoma G3, serous carcinoma, and clear-cell and undifferentiated carcinomas (p < 0.001, p = 0.003, and p = 0.019, respectively). The details of the sites of recurrence based on histological analysis are shown in Table 3.

**Table 3:** Patient and tumor characteristics

Histology and sites of recurrence																			
Stage IC		Recurrence			Multiple			VSM			HM			LNM			DM		
	Numbers	Numbers	%	p value	Numbers	%	p value	Numbers	%	p value	Numbers	%	p value	Numbers	%	p value	Numbers	%	p value
Endometrioid carcinoma Grade 3	16	1	6.3	0.332	1	6.3	–	1	6.3	–	0	0	0.219	1	6.3	0.978	0	0	0.219
Carcinosarcomas	11	2	18.2	–	0	0	0.398	0	0	0.398	1	9.1	–	0	0	0.388	1	9.1	–
Mixed type carcinoma	5	0	0	0.301	0	0	0.566	0	0	0.566	0	0	0.486	0	0	0.558	0	0	0.486
Serous carcinoma	31	3	9.7	0.454	1	3.2	0.625	0	0	0.159	0	0	0.089	2	6.5	–	2	6.5	0.77
Clear-cell and Undifferentiated carcinoma	10	0	0	0.156	0	0	0.42	0	0	0.42	0	0	0.328	0	0	0.41	0	0	0.326

Stage IIC-H		Recurrence			Multiple			VSM			HM			LNM			DM		
	Numbers	Numbers	%	p value	Numbers	%	p value	Numbers	%	p value	Numbers	%	p value	Numbers	%	p value	Numbers	%	p value
Endometrioid carcinoma Grade 3	79	26	32.9	0.568	6	7.6	0.125	6	7.6	0.521	6	7.6	0.125	1	1.3	< 0.001*	5	6.3	0.346
Carcinosarcomas	97	21	21.6	0.044*	11	11.3	0.39	6	6.2	0.292	13	13.4	0.614	12	12.4	0.089	10	10.3	–
Mixed type carcinoma	42	16	38.1	–	7	16.7	–	2	4.8	0.279	7	16.7	–	10	23.8	–	3	7.1	0.556
Serous carcinoma	86	21	24.4	0.109	4	4.7	0.022*	9	10.5	–	6	7	0.088	5	5.8	0.003*	5	5.8	0.268
Clear-cell and Undifferentiated carcinoma	29	8	27.5	0.357	1	3.4	0.083	2	6.9	0..572	4	13.8	0.742	1	3.4	0.019*	3	10.3	0.995

*VSM* Vaginal stump metastases, *DM* Disseminated metastases, *LNM* Lymph node metastases, *HM* Hematogenous metastases

^*^p < 0.05

### PFS and OS

**Fig. 1:**
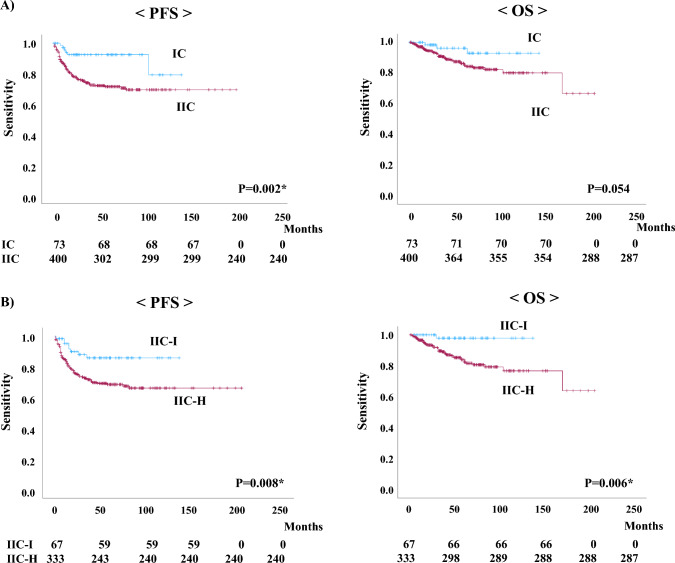
**A** Comparison of progression-free survival (PFS) and overall survival (OS) in patients with 2023 International Federation of Gynecology and Obstetrics (FIGO) stage IC and IIC endometrial cancer. **B** Comparison of PFS and OS in patients with 2023 FIGO stage IIC-intermediate risk (IIC-I), and IIC-high risk (IIC-H) endometrial cancer

Regarding PFS and OS, patients in the IIC group had a significantly higher progression rate than patients in the IC group (PFS: p = 0.002, and OS: p = 0.054) (Fig. 1A). And, the PFS and OS were significantly worse in patients with FIGO 2023 stage IIC-H EC than in those with FIGO 2023 stage IIC-I EC (PFS: p = 0.008 and OS: p = 0.006) (Fig. 1B).

### PFS and OS for each treatment modality

**Fig. 2:**
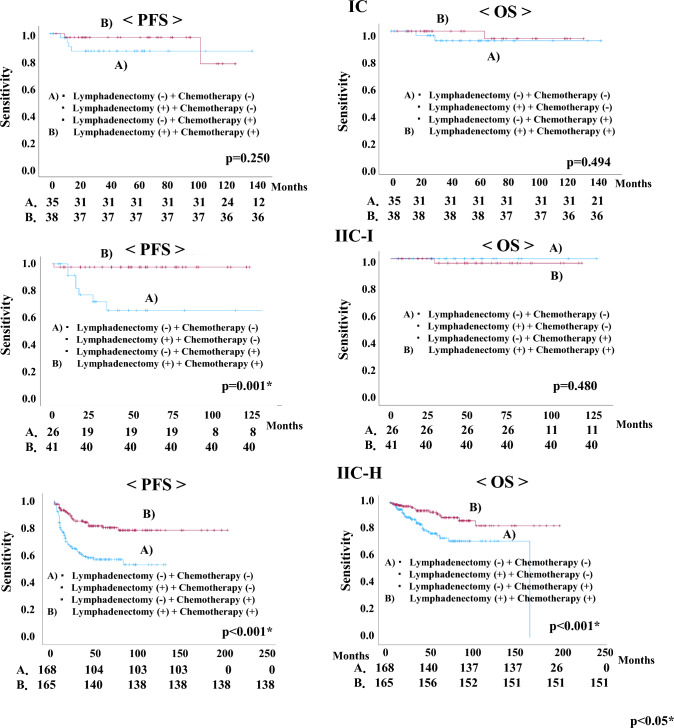
Comparison of progression-free survival (PFS) and overall survival (OS) in patients with 2023 International Federation of Gynecology and Obstetrics (FIGO) stage IC, IIC-intermediate risk (IIC-I), and IIC-high risk (IIC-H) endometrial carcinoma in two treatment groups: **A** No lymphadenectomy and no adjuvant chemotherapy, Lymphadenectomy and no adjuvant chemotherapy, and No lymphadenectomy and adjuvant chemotherapy; or **B** Lymphadenectomy and adjuvant chemotherapy

**Fig. 3:**
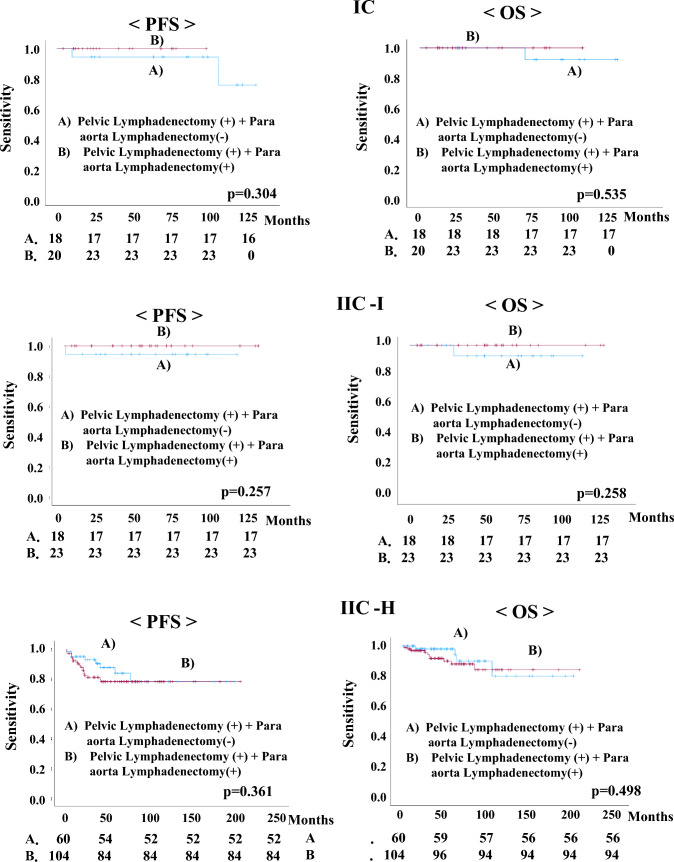
Comparison of progression-free survival (PFS) and overall survival (OS) in patients with 2023 FIGO stage IC, IIC-intermediate risk (IIC-I), and IIC-high risk (IIC-H) endometrial carcinoma who received adjuvant chemotherapy after pelvic lymphadenectomies with or without para-aorta lymphadenectomies

Regarding the treatment of patients in the IC, IIC-I, and IIC-H groups were divided into two groups. PFS and OS rates were examined in the following two groups: A) No lymphadenectomy and no adjuvant chemotherapy, lymphadenectomy and no adjuvant chemotherapy, and no lymphadenectomy and adjuvant chemotherapy, or B) lymphadenectomy and adjuvant chemotherapy. In the IIC-I group, lymphadenectomy and adjuvant chemotherapy had a significantly better PFS than other treatment modalities (p = 0.001). In the IIC-H group, lymphadenectomy and adjuvant chemotherapy had better prognoses regarding both PFS and OS than the other treatment modalities (PFS, p < 0.001; OS, p < 0.001) (Fig. 2). Regarding patients who received lymphadenectomies and chemotherapy, we investigated the PFS and OS rates with pelvic lymphadenectomies alone or pelvic lymphadenectomy combined with para-aortic lymphadenectomies in the IC, IIC-I, and IIC-H groups. However, combination para-aortic lymphadenectomy did not improve the PFS or OS rates in any of the groups (Fig. 3).

### Recurrence sites and histological types in different treatment modalities

We investigated the recurrence sites in the IIC-I and IIC-H groups. In the IIC-I group, lymphadenectomies and chemotherapy significantly reduced the incidence of VSM compared with other treatment modalities (p = 0.009). In the IIC-H group, lymphadenectomies and chemotherapy significantly reduced the incidence of VSM and LNM compared with other treatment modalities (p < 0.001 and p = 0.008, respectively). Lymphadenectomies and chemotherapy significantly reduced the recurrence of endometrioid adenocarcinoma G3, carcinosarcoma, and serous carcinoma compared with other treatment modalities (p = 0.024, p = 0.014, and p = 0.048, respectively). In mixed-type, clear-cell, and undifferentiated carcinomas, the prognosis was not affected by the treatment modality used (Table 4A).

**Table 4:** Stage IIC recurrence sites and histologies by treatment modalities

All cases					
IIC-I					
Recurrence site					
	Non recurrence	Non recurrence	Recurrence	Recurrence	
	Lymph (–); Chemo (–) Lymph ( +); Chemo (–) Lymph (–); Chemo ( +)	Lymph ( +); Chemo ( +)	Lymph (–); Chemo (–) Lymph (+); Chemo (–) Lymph (–); Chemo (+)	Lymph (+); Chemo (+)	p value
Vaginal stump metastases	22	41	4	0	0.009*
Disseminated metastases	24	41	2	0	0.071
Lymph node metastases	24	40	1	1	0.719
Hematogenous metastases	24	41	2	0	0.071

IIC-H					
Recurrence site					
	Non recurrence	Non recurrence	Recurrence	Recurrence	
	Lymph (–); Chemo (–) Lymph (+); Chemo (–) Lymph (–); Chemo (+)	Lymph (+); Chemo (+)	Lymph (–); Chemo (–) Lymph (+); Chemo (–) Lymph (–); Chemo (+)	Lymph (+); Chemo (+)	p value
Vaginal stump metastases	149	161	20	3	< 0.001*
Disseminated metastases	149	154	20	10	0.067
Lymph node metastases	158	157	22	7	0.008*
Hematogenous metastases	153	150	16	14	0.766

Histology					
	Non recurrence	Non recurrence	Recurrence	Recurrence	
	Lymph (–); Chemo (–) Lymph (+); Chemo (–) Lymph (–); Chemo (+)	Lymph (+); Chemo (+)	Lymph(–); Chemo(–) Lymph(+); Chemo(–) Lymph(–); Chemo(+)	Lymph (+); Chemo (+)	p value
Endometrioid adenocarcinoma G3	25	40	10	4	0.024*
Carcinosarcomas	32	32	25	8	0.014*
Mixed type carcinoma	10	16	9	7	0.261
Serous carcinoma	31	33	16	6	0.048*
Clear-cell and undifferentiated carcinoma	7	13	5	4	0.298

Lymphadnectomy					
IIC-I					
Recurrence site					
	Non recurrence	Non recurrence	Recurrence	Recurrence	
	Chemotherapy (–)	Chemotherapy (+)	Chemotherapy (–)	Chemotherapy ( +)	p value
Vaginal stump metastases	1	40	0	0	-
Disseminated metastases	1	40	0	0	-
Lymph node metastases	1	39	0	1	0.872
Hematogenous metastases	1	40	0	0	-

IIC-H					
Recurrence site					
	Non recurrence	Non recurrence	Recurrence	Recurrence	
	Chemotherapy (–)	Chemotherapy (+)	Chemotherapy (–)	Chemotherapy ( +)	p value
Vaginal stump metastases	31	162	2	3	0.156
Disseminated metastases	27	155	6	10	0.019*
Lymph node metastases	31	158	2	7	0.647
Hematogenous metastases	28	151	5	14	0.232

Histology					
	Non recurrence	Non recurrence	Recurrence	Recurrence	
	Chemotherapy (–)	Chemotherapy (+)	Chemotherapy (–)	Chemotherapy ( +)	p value
Endometrioid adenocarcinoma G3	7	41	2	4	0.245
Carcinosarcomas	7	32	3	8	0.495
Mixed type carcinoma	1	16	4	7	0.033*
Serous carcinoma	3	33	4	6	0.013*
Clear-cell and Undifferentiated carcinoma	2	13	0	4	0.44

*G* grade, *Lymph* lymphadnectomy, *Chemo* chemotherapy

^*^p < 0.05

When only the groups that underwent lymphadenectomies were compared, there was a significant reduction in DM (p = 0.019) in the IIC-H group. Regarding histological types, mixed-type carcinoma and serous carcinomas had significantly fewer recurrences in the IIC-H group (p = 0.033 and p = 0.013, respectively) (Table 4B).

### Prognostic factors for PFS and OS

The correlations between clinical factors, PFS, and OS were assessed through univariate and multivariate analyses in patients in the IIC-H group who underwent lymphadenectomies (Table 5). In the univariate analysis, age (p < 0.001), histology (p = 0.012), peritoneal cytology (p < 0.001), and treatment modality (p < 0.001) were significantly associated with worse PFS in the IIC-H group. In the multivariate analysis, age (p = 0.003), histology (p = 0.014), LVS involvement (p = 0.007), peritoneal cytology (p < 0.001), and treatment modality (p < 0.001) were significantly associated with worse PFS in patients in the IIC-H group. In the univariate analysis, age (p < 0.001), peritoneal cytology (p = 0.023), and treatment modality (p = 0.001) were significantly associated with worse OS in the IIC-H group. In the multivariate analysis, age (p < 0.001), LVS involvement (p = 0.012), peritoneal cytology (p = 0.015), and treatment modality (p = 0.031) were significantly associated with worse OS in patients in the IIC-H group (Table 5A).

**Table 5:** Prognostic factors for PFS and OS selected through Cox’s univariate and multivariate analyses

IIC-H						
PFS	Univariate analysis			Multivariate analysis		
	Exp (B)	95% CI	Cox’s test	Exp (B)	95% CI	Cox’s test
			p value			p value
Age, years (≥ 70)	2.631	1.729–4.005	< 0.001*	2.021	1.277–3.200	0.003*
BMI, kg/m^2^ (≥ 30.0)	0.959	0.464–1.979	0.9	1.04	0.496–2.180	0.913
Myometrial invasion (> 50%)	1.107	0.736–1.665	0.624	1.281	0.808–2.032	0.292
Histology (non-endometrioid carcinoma)	2.127	1.183–3.824	0.012*	2.261	1.180–4.333	0.014*
LVS involvement (present)	1.462	0.973–2.195	0.067	1.791	1.169–2.745	0.007*
Peritoneal cytology (present)	2.389	1.427–3.999	< 0.001*	2.571	1.500–4.410	< 0.001*
Treatment (no lymphadnectomy or no chemotherapy)	2.786	1.786–4.346	< 0.001*	2.318	1.441–3.729	< 0.001*

OS	Univariate analysis			Multivariate analysis		
	Exp (B)	95% CI	Cox’s test	Exp(B)	95% CI	Cox’s test
			p value			p value
Age, years (≥ 70)	4.246	2.270–7.943	< 0.001*	3.499	1.770–6.918	< 0.001*
BMI, kg/m^2^ (≥ 30.0)	0.454	0.110–1.875	0.275	0.43	0.103–1.799	0.248
Myometrial invasion (> 50%)	1.292	0.717–2.326	0.394	1.231	0.635–2.384	0.538
Histology (non-endometrioid carcinoma)	1.918	0.857–4.294	0.113	2.13	0.871–5.211	0.098
LVS involvement (present)	1.729	0.967–3.090	0.065	2.21	1.192–4.097	0.012*
Peritoneal cytology (present)	2.341	1.126–4.866	0.023*	2.639	1.232–5.652	0.013*
Treatment (no lymphadnectomy or no chemotherapy)	2.874	1.523–5.423	0.001*	2.105	1.070–4.141	0.031*

B) Lymphadenectomy						
IIC-H						
PFS	Univariate analysis			Multivariate analysis		
	Exp (B)	95% CI	Cox’s test	Exp(B)	95% CI	Cox’s test
			p value			p value
Age, years (≥ 70)	2.867	1.551–5.298	< 0.001*	2.629	1.347–5.130	0.005*
BMI, kg/m^2^ (≥ 30.0)	0.34	0.047–2.471	0.286	0.354	0.048–2.610	0.308
Myometrial invasion (> 50%)	1.026	0.558–1.885	0.935	0.956	0.488–1.870	0.895
Histology (non-endometrioid carcinoma)	1.908	1.052–3.460	0.033*	2.145	1.081–4.256	0.029
LVS involvement (present)	1.441	0.787–2.642	0.237	2.957	1.434–6.097	0.003*
Peritoneal cytology (present)	4.64	2.402–8.960	< 0.001*	5.235	2.646–10.359	< 0.001*
Treatment (no chemotherapy)	2.457	1.277–4.729	0.007*	2.994	1.453–6.170	0.003*

OS	Univariate analysis			Multivariate analysis		
	Exp (B)	95% CI	Cox’s test	Exp(B)	95% CI	Cox’s test
			p value			p value
Age, years (≥ 70)	5.149	2.153–12.314	< 0.001*	5.074	1.931–13.333	< 0.001*
BMI, kg/m^2^ (≥ 30.0)	0.045	0–145.916	0.452	0	0	0.984
Myometrial invasion (> 50%)	1.014	0.425–2.418	0.975	0.707	0.265–1.881	0.487
Histology (non-endometrioid carcinoma)	1.652	0.725–3.764	0.232	1.59	0.587–4.305	0.361
LVS involvement (present)	1.211	0.514–2.855	0.661	2.42	0.867–6.748	0.091
Peritoneal cytology (present)	3.322	1.201–9.187	0.021*	3.838	1.336–11.023	0.012*
Treatment (no chemotherapy)	2.294	0.925–5.691	0.073	1.861	0.710–4.878	0.207

*BMI* Body mass index, *PFS* progression-free survival, *OS* overall survival, *CI* confidence interval

^*^p < 0.05

We performed univariate and multivariate analyses of PFS and OS for patients in the IIC-H group who underwent lymphadenectomies. In the univariate analysis, age (p < 0.001), histology (p = 0.013), peritoneal cytology (p < 0.001), and treatment modality (p = 0.007) were significantly associated with worse PFS after lymphadenectomy in the IIC-H group. In the multivariate analysis, age (p = 0.003), LVS involvement (p = 0.003), peritoneal cytology (p < 0.001), and treatment modality (p = 0.003) were significantly associated with worse PFS in patients in the IIC-H group who underwent lymphadenectomies. In the univariate analysis, age (p < 0.001) and peritoneal cytology (p = 0.021) were significantly associated with worse OS in patients in the IIC-H group who underwent lymphadenectomies. In the multivariate analysis, age (p < 0.001) and LVS involvement (p = 0.012) were significantly associated with worse OS in patients in the IIC-H group who underwent lymphadenectomies (Table 5B).

## Discussion

PFS and OS rates were significantly worse in the IIC-H group than in the IC and IIC-I groups. In patients in the IIC-H group, lymphadenectomies and chemotherapy resulted in better prognoses regarding both PFS and OS than the other treatment modalities. These findings suggest that lymphadenectomies and chemotherapy effectively reduced VSM and LNM in FIGO 2023 stage IIC-H EC. Furthermore, not undergoing lymphadenectomies or chemotherapy is an independent predictor of recurrence and survival in FIGO 2023 stage IIC-H EC.

The FIGO staging classification for endometrial cancer (FIGO 2023) has been completely revised to include histopathological and molecular genetic information, in addition to traditional anatomical progression, marking a major shift toward improving prognostic prediction and individualizing treatment [3]. Even at the same stage of progression, the prognoses of typical endometrioid carcinoma and aggressive histologic carcinoma differ significantly, making it easier to predict prognosis using the new FIGO 2023 stage classification. DNA polymerase ε (POLE), mismatch repair (MMR), and p53 are important molecular subtypes that affect staging and prognostic prediction [3], but since this is a multicenter study based on data from 2003, the POLE, MMR, and p53 molecular subtypes were not analyzed. In the future, molecular subtypes will be important for better staging and prognostic prediction. Therefore, Incorporating molecular subtypes into FIGO 2023 IC and IIC may further enhance staging and prognosis.

Recurrence is common in early-stage aggressive histological carcinomas, and treatment methods have been controversial. To date, several trials have been conducted to determine whether or not to perform lymph node dissections and how to select postoperative treatment methods. The ASTEC and Italian studies reported that prognoses were not affected by whether lymphadenectomies were performed [4, 5], whereas the SEPAL study reported that prognoses were significantly better in patients who underwent pelvic lymphadenectomies and para-aortic lymphadenectomies [6]. Various postoperative treatments have been attempted, including chemotherapy, radiation therapy, and chemoradiotherapy (GOG249 and PORTEC-3) [7–9]. However, surgical procedures and adjuvant treatments often vary, and there is no established gold-standard treatment yet. The NCCN 2024 guidelines recommend total hysterectomy, bilateral salpingo-oophorectomy, omentectomy, staging surgery, and systemic treatments such as adjuvant chemotherapy for early-stage aggressive histological carcinomas in patients with EC. Radiotherapy is often listed as an option in the NCCN guidelines for the treatment of early-stage aggressive histological carcinomas in patients with EC [10]. The JSGO guidelines for EC recommend total hysterectomy, bilateral salpingo-oophorectomy, omentectomy, pelvic/para-aortic lymphadenectomy, and systemic treatments, such as adjuvant chemotherapy [2].

In the present study, FIGO 2023 stage IIC, which has myometrial invasion, showed a significantly higher recurrence rate and a higher tendency for mortality than stage IC, which has no myometrial invasion in early-stage aggressive histological carcinomas. Patients with high-risk FIGO 2023 stage IIC EC had significantly higher recurrence and mortality rates than those with intermediate-risk FIGO 2023 stage IIC EC. In the FIGO 2023 stage IIC-H group, lymphadenectomy and chemotherapy had better prognoses regarding both recurrence and survival rates than other treatment modalities. Lymphadenectomy and chemotherapy effectively reduced VSM and LNM in the IIC-H group, and not receiving lymphadenectomies or chemotherapy were independent predictors of recurrence and poor prognoses in the IIC-H group. However, there is no evidence regarding the efficacy of para-aortic lymphadenectomies in patients with FIGO 2023 stage IC, IIC-I, or IIC-H EC. In patients who underwent lymphadenectomies, PFS was influenced by age, non-endometrioid histology, LVS involvement, peritoneal cytology, and adjuvant chemotherapy, whereas OS was influenced by age, LVS involvement, and peritoneal cytology. Non-endometrioid carcinoma and LVS involvement are associated with poor prognoses regarding recurrence-free survival, while non-endometrioid carcinoma is associated with poor prognoses regarding OS in patients with FIGO 2023 stage IIC EC who undergo lymphadenectomies [11]. Furthermore, LVS and myometrial invasion are associated with both disease-free survival and OS in patients with FIGO 2023 stage IIC EC who undergo lymphadenectomies [12]. Aggressive histological cancer types are more prevalent in older adult patients and in those with comorbidities; more than 40% of the patients in this study were over 70 years of age. In the IIC group, patients aged ≥ 80 years accounted for 13.3% of cases. Consequently, many patients were unable to undergo chemotherapy after their lymphadenectomies, and new treatments are needed.

The limitations of this study are as follows. First, it was a retrospective analysis. Second, it was a small study. Large-scale prospective studies are required to investigate the clinical significance of FIGO 2023 stage IIC EC further.

In summary, this study revealed that lymphadenectomies and chemotherapy as treatment modalities led to better prognoses regarding both recurrence and survival in patients with FIGO 2023 stage IIC- high-risk EC.

## Data Availability

The datasets generated and analyzed during the current study are available from the corresponding author upon reasonable request.
